# T232. EFFICACY AND SAFETY OF ANTIDEPRESSANT AUGMENTATION OF ANTIPSYCHOTICS IN SCHIZOPHRENIA

**DOI:** 10.1093/schbul/sby016.508

**Published:** 2018-04-01

**Authors:** Britta Galling, Christoph Correll

**Affiliations:** 1 The Zucker Hillside Hospital; 2 The Zucker Hillside Hospital, Hofstra North Shore LIJ School of Medicine

## Abstract

**Background:**

Although antidepressants are commonly used in patients with schizophrenia, meta-analytic guidance on the efficacy and safety of antidepressant augmentation evaluated as a single clinical strategy in patients with insufficient response to antipsychotic monotherapy is missing.

**Methods:**

Systematic literature search of PubMed/MEDLINE/PsycINFO/Cochrane Library without language restrictions from database inception until 07/20/2015 for randomized, double-blind, efficacy-focused trials comparing adjunctive antidepressants vs placebo to antipsychotics in schizophrenia. Random effects meta-analysis in accordance with the Preferred Reporting Items for Systematic Reviews and Meta-Analyses (PRISMA) standard to evaluate the efficacy and safety of antidepressant augmentation of antipsychotics in schizophrenia.

**Results:**

In 48 studies (n=2192, duration=10.2 ± 7.6 weeks), antidepressant augmentation outperformed placebo regarding total symptom reduction (studies=28, n=1249, standardized mean difference (SMD)=-0.36, 95% confidence interval (CI)=-0.57, -0.15, p=0.001), driven by negative (studies=32; n=1384, SMD=-0.25, 95%CI=-0.44, -0.06, p=0.010), but not positive (p=0.284) or general (p=0.118) symptom reduction. Significant improvements extended to core negative symptoms avolition/apathy (SMD=-0.54, 95%CI=-0.84, -0.24, p<0.001) and anhedonia/asociality (studies=8, n=284, SMD=-0.50, 95%CI=-0.90, -0.10, p=0.013). In predefined subgroup-analyses, superiority regarding negative symptoms was confirmed in studies augmenting first-generation antipsychotics (FGAs) (studies=10, n=433, SMD=-0.42, 95%CI=-0.76, -0.08, p=0.016), but not second-generation antipsychotics (studies=13, n=452, p=0.385). Uniquely, superiority in total symptom reduction by noradrenergic-and-specific-serotonergic-antidepressants (SMD=-0.72, 95%CI=-1.24, -0.20, p=0.007) was not driven by negative (p=0.467), but by positive symptom reduction (SMD=-0.43, 95%CI=-0.78, -0.09, p=0.013). Antidepressants did not improve depressive symptoms more than placebo (studies=24, n=1111, p=0.207). Bupropion was superior to placebo regarding smoking cessation (studies=7, n=327, RR=2.75, 95%CI=1.60–4.72, p<0.001, number-needed-to-treat (NNT)=6). Except for more dry mouth (RR=1.57, 95%CI=1.04–2.36, p=0.03) and dizziness (RR=2.01, 95%CI=1.06–3.82, p=0.032), antidepressants were not associated with more adverse effects or all-cause/specific-cause discontinuation than placebo.

**Discussion:**

For schizophrenia patients on stable antipsychotic treatment adjunctive antidepressants are effective for total and particularly negative symptom reduction, and bupropion helps smoking cessation. However, effects are small-to-medium, differ across individual antidepressants, and negative symptom improvement seems restricted to the augmentation of FGAs.

